# Surgical advances in the management of brain metastases

**DOI:** 10.1093/noajnl/vdab130

**Published:** 2021-11-27

**Authors:** Patrick R Ng, Bryan D Choi, Manish K Aghi, Brian V Nahed

**Affiliations:** 1 Department of Neurosurgery, Massachusetts General Hospital, Harvard Medical School, Boston, Massachusetts, USA; 2 Department of Neurosurgery, University of California San Francisco, San Francisco, CA, USA

**Keywords:** brachytherapy, intraoperative neuroimaging and neuromonitoring, fluorescent-guided neurosurgery, minimally invasive neurosurgery, supramarginal surgery

## Abstract

As the epidemiological and clinical burden of brain metastases continues to grow, advances in neurosurgical care are imperative. From standard magnetic resonance imaging (MRI) sequences to functional neuroimaging, preoperative workups for metastatic disease allow high-resolution detection of lesions and at-risk structures, facilitating safe and effective surgical planning. Minimally invasive neurosurgical approaches, including keyhole craniotomies and tubular retractors, optimize the preservation of normal parenchyma without compromising extent of resection. Supramarginal surgery has pushed the boundaries of achieving complete removal of metastases without recurrence, especially in eloquent regions when paired with intraoperative neuromonitoring. Brachytherapy has highlighted the potential of locally delivering therapeutic agents to the resection cavity with high rates of local control. Neuronavigation has become a cornerstone of operative workflow, while intraoperative ultrasound (iUS) and intraoperative brain mapping generate real-time renderings of the brain unaffected by brain shift. Endoscopes, exoscopes, and fluorescent-guided surgery enable increasingly high-definition visualizations of metastatic lesions that were previously difficult to achieve. Pushed forward by these multidisciplinary innovations, neurosurgery has never been a safer, more effective treatment for patients with brain metastases.

Brain metastases (BMs) are the most common type of intracranial tumor in adults, occurring about 10 times more frequently than primary malignant brain tumors.^1^ Population-based studies estimate that 8.5–9.6% of cancer patients will develop a BM,^2,3^ while autopsy studies suggest that approximately 25% of people who die of cancer had developed metastatic disease to the brain.^4,5^ Incidence rates of BMs are difficult to assess since no national registries exist specifically for patients with brain metastases, and current estimates likely underestimate the true burden of disease.^6^ As cancer treatment, diagnosis, and surveillance improve, incidence rates will continue to rise.^1,6^ Neurosurgery is an essential tool in the therapeutic arsenal against brain metastases and has been shown to improve survival and quality of life.^7,8^ Given the growing epidemiological and clinical burden of brain metastases, advancements in surgical management are imperative. This review will cover recent innovations in neurosurgical techniques and intraoperative considerations for the treatment of brain metastases (Table 1).

**Table 1.** Surgical Innovations for Brain Metastases

Surgical Innovations for Brain Metastases	
Preoperative Workup	Neuroimaging (CT, MRI, functional imaging)
Surgical Approach	Minimally invasive craniotomies
	Tubular retractors
	Supramarginal resection
	Brachytherapy
Intraoperative Augmentation	Neuronavigation
	Intraoperative ultrasound
	Intraoperative brain mapping
	Endoscope
	Exoscope
	Fluorescence-guided surgery

## Preoperative Considerations

The preoperative workup of lesions suggestive of brain metastases centers on neuroimaging. Computed tomography (CT) rules out neurosurgical emergencies, provides superior visualization of bony details, particularly if metastases involve the calvarium, and is used for patients with MRI contraindications.^9^ Contrast-enhanced magnetic resonance imaging (MRI) offers superior sensitivity in detecting metastases, especially when the lesions are small or located in the posterior fossa, frontotemporal region, and cortex.^9–11^ BMs do not have any pathognomonic features on CT or MRI, but post-contrast T1 enhancement, ring-enhancement, spherical shape, and multiple lesions are suggestive of metastatic disease.^9^ Other imaging modalities, such as diffusion-weighted imaging (DWI), susceptibility-weighted imaging (SWI), diffusion tensor imaging (DTI), MR perfusion imaging, ^18^F-2-fluoro-2-deoxy-D-glucose (FDG) positron emission tomography (PET), amino acid PET, and MR spectroscopy, are actively under investigation for diagnosing metastases, differentiating metastases from similar-appearing lesions, and identifying the primary tumor type.^9^ For lesions located in eloquent areas of the brain, preoperative imaging may include functional MRI (fMRI), transcranial magnetic stimulation (TMS), and/or DTI. all of which have been shown to improve surgical outcomes.^12–14^

If a diagnosis is required and surgical resection would not be safe, a suspected lesion should be biopsied.^15^ Routine hematoxylin-eosin (H&E) staining of surgical specimens differentiates metastases from other lesions, while immunohistochemical markers are utilized if H&E findings are equivocal.^16^ Molecular analysis of samples can identify the tissue of origin in cases where whole-body imaging and H&E staining fail to do so, or lineage markers and biomarkers, both of which can impact treatment strategies such as selection of targeted agents and eligibility for clinical trials.^17^ Given recent findings that BMs harbor molecular differences compared to their respective primary tumors,^18^ securing BM tissue samples through biopsies or surgery for molecular analysis will become more common to assist in clinical decision-making.

The main objectives of surgery are to acquire tissue for diagnosis, reduce symptomatic mass effect and vasogenic edema, definitively treat local lesions with improved quality of life, and prolong overall survival when combined with adjuvant radiation therapy.^19^ Two randomized controlled trials from the early 1990s established the overall survival and functional benefits of surgery with adjuvant radiotherapy over radiotherapy alone.^20,21^ For patients with multiple brain metastases, retrospective studies have shown that resecting up to three metastases offers survival rates comparable to those of patients who underwent resection of a single BM.^22–24^ Surgery alone is not sufficient for local control of BMs and therefore must be complemented with either whole-brain radiation therapy (WBRT) or stereotactic radiosurgery (SRS), though SRS is preferred when safe and especially for low tumor volumes.^15^ The 2019 Congress of Neurological Surgeons (CNS) guidelines for the treatment of adults with metastatic brain tumors can be found in Table 2.^25^

**Table 2.** 2019 Congress of Neurological Surgeons (CNS) Guidelines on the Role of Surgery in the Management of Adults with Metastatic Brain Tumors

Target Population	Question	Recommendations
Adult patients with newly diagnosed metastatic brain tumors, excluding radiosensitive tumor histologies.	Should patients with newly diagnosed metastatic brain tumors undergo surgery, stereotactic radiosurgery (SRS), or whole brain radiation therapy (WBRT)?	*Level 1:* Surgery + WBRT is recommended as first-line treatment in patients with single brain metastases with favorable performance status and limited extracranial disease to extend overall survival, median survival, and local control. *Level 3:* Surgery + SRS is recommended to provide survival benefit in patients with metastatic brain tumors *Level 3:* Multimodal treatments including either surgery + WBRT + SRS boost or surgery + WBRT are recommended as alternatives to WBRT + SRS in terms of providing overall survival and local control benefits.
	Should patients with newly diagnosed metastatic brain tumors undergo surgical resection followed by WBRT, SRS, or another combination of these modalities?	*Level 1:* Surgery + WBRT is recommended as superior treatment to WBRT alone in patients with single brain metastases. *Level 3:*Surgery + SRS is recommended as an alternative to treatment with SRS alone to benefit overall survival. *Level 3:*It is recommended that SRS alone be considered equivalent to surgery + WBRT.
Adult patients diagnosed with recurrent, non-radiosensitive metastatic brain tumors.	Should patients with recurrent metastatic brain tumors undergo surgical resection?	*Level 3:* Craniotomy is recommended as a treatment for intracranial recurrence after initial surgery or SRS.

Key clinical questions and levels of each recommendation, which are directly linked to Class I, II, or III evidence, are included in the table. Class I evidence is extrapolated to Level 1 recommendations or lower. Class II evidence is extrapolated to Level 2 recommendations or lower. Class III evidence only yields Level 3 recommendations. The table is adapted from Nahed et al. 2019 with permission.^25^

## Surgical Approach

### Minimally Invasive Craniotomy

Improvements in preoperative diagnostic imaging and intraoperative illumination devices have facilitated the miniaturization of cranial approaches, from Dandy’s “macrosurgical” craniotomies to Yasargil’s microneurosurgery to today’s minimally invasive techniques.^26^ While standard craniotomy approaches can effectively access various intracranial lesions, minimally invasive approaches can also be tailored uniquely to the target lesion.^27^ Conventional craniotomies typically produce openings that are larger than the target, whereas keyhole craniotomies can create openings (~2–5 cm in diameter) smaller than the target with complete exposure achieved by subtending the angles of approach.^26–28^ Keyhole approaches limit brain exploration and retraction and embody the operative philosophy of preserving as much normal tissue as possible while achieving sufficient exposure and maximal resection. Studies have shown that keyhole approaches minimize soft tissue and bone trauma, decrease postoperative complications, and improve cosmetic results.^29^ Due to a more restricted surgical corridor and limited visual control, keyhole approaches are typically augmented with endoscopic devices, special tube-shaft microinstruments, and intraoperative imaging, monitoring, and mapping.^26^ Since the surgical pathway cannot be changed during surgery, meticulous preoperative planning is paramount.^26^

Keyhole approaches can enable safe and effective resection of brain metastases. In Tobler and Stanley,^30^ stereotactic-guided keyhole craniotomies enabled gross total resection (GTR) in 100% of 14 patients with metastatic tumors located in eloquent cortical regions. Moreover, 88% of patients experienced significant alleviation or elimination of their preoperative neurologic deficits.^30^ Phang et al.^31^ achieved complete resection in 85% of 35 patients with brain metastases with a range of tumor volumes and locations, including the posterior fossa, all cortical lobes, intraventricular regions, and the basal ganglia. Median survival of these patients was comparable to that of the literature, and compared to controls in the same cohort, patients who underwent minimally invasive keyhole surgeries experienced shorter mean length of stay.^31^ Lastly, Baker et al.^32^ showed that patients with multiple (2 to 10+) brain metastases in diverse locations (ie, the cortical lobes, the posterior fossa, and the thalamus) may undergo 2 to 4 simultaneous keyhole craniotomies and resections with survival outcomes and surgical risks comparable to patients undergoing resection of single brain metastases. These patients also experienced improvements in Karnofsky Performance Scale (KPS) scores during the early postoperative period and were successfully weaned from steroids.^32^

The supraorbital (SO) “eyebrow” craniotomy is a keyhole modification of the standard pterional approach and is especially useful for metastases located in the orbitofrontal surface and frontal pole.^33^ Similar to other minimally invasive techniques, the SO approach minimizes soft tissue/bone trauma and brain exposure to non-physiologic surroundings, minimizes brain retraction, decreases the time between skin incision to dural opening (~10 min), decreases approach-related morbidity, improves cosmetic results, and shortens hospitalization.^34^ In Reisch et al.,^35^ 77% of 375 patients who underwent a SO craniotomy experienced no follow-up pain, and 84% were very pleased with the cosmetic outcome. In Eroglu et al.,^36^ 84.6% of 13 BM cases achieved GTR with the SO approach, and 92.3% of these patients were highly satisfied with the cosmetic result.

Minimally invasive techniques can be applied to any standard craniotomy.^28^ For example, Bonney *et al.* showed that the supracerebellar-infratentorial (SCIT) approach, once thought to require a large craniotomy extending inferiorly to the rim of the foramen magnum, can be achieved with a 2.5 cm keyhole opening.^37^ This approach enabled near-total resection of a pontine lung metastasis in the pineal region.^37^ As data on the safety and efficacy of minimally invasive methods continues to emerge and as surgeons gain experience with these techniques, keyhole craniotomies will become a preferred approach for select BM patients.

### Tubular Retractors

Resection of deep intracranial lesions depends on proper visualization and access along the surgical corridor, which requires retraction of surrounding structures. Though handheld blade retractors and mounted devices are commonly used, these techniques can exert prolonged, focal pressure on the brain parenchyma, which has been shown to compromise vascular flow, induce local ischemia, and cause direct tissue injury.^38^ First introduced by Kelly et al.,^39^ tubular retractors establish surgical corridors by displacing the parenchyma with blunt tips and evenly distributing radial force to the surrounding tissue. Initially designed to attach to a stereotaxic frame, tubular retractors are now typically positioned with frameless neuronavigational systems.^40^ Preoperative imaging, including DTI, identifies pathways that minimize the disruption of white matter tracts.^41^ Though postoperative DWI/ADC imaging has shown that tubular retraction can still cause cytotoxic edema and cellular damage,^42^ complication rates with tubular retractors have been shown to be lower than with traditional paddle retractors.^43^ The three main tubular retractors that have been studied for the resection of deep intracranial lesions are the ViewSite Brain Access System (VBAS), the BrainPath tubular retraction system, and the Minimal Exposure Tubular Retractor system (METRx), though the METRx was originally designed for minimally invasive spinal surgery and is not approved for intracranial use.^43^ There is no difference in patient outcomes between BrainPath and VBAS.^44^

Tubular retractors have been implemented for the resection of deep-seated metastases. Depending on the surgeon’s experience, operative microscopes (OMs),^45^ exoscopes,^44,46,47^ endoscopes,^41,45^ intraoperative MRI,^48^ and intraoperative ultrasound^49^ may be used with tubular-retractor-assisted resection of BMs. Though most commonly utilized for subcortical and periventricular lesions, tubular retractors can also facilitate high-efficacy, low-morbidity resection of brain metastases in the posterior fossa.^47,49^ Furthermore, the physical characteristics of the BM determine the efficacy of tubular retractors. Softer metastases, such as breast and melanoma, are favored, allowing depth cannulation into the tumor and inside-out resection.^41,45,46^ Firmer lesions, such as metastatic sarcoma, require surface cannulation to avoid displacement of the tumor, while hemorrhage-prone BMs, such as renal cell carcinoma, are less amenable to the piecemeal resection executed with tubular retractors.^41,45^ In retrospective clinical studies, high rates of GTR (73% to 100%) of deep-seated brain metastases have been achieved with BrainPath, VBAS, and METRx.^40,41,45–47^ Tubular-retractor-assisted biopsy has also been studied as an alternative to stereotactic needle biopsy and has demonstrated superior tissue yield without the need for reoperation.^50^

### Supramarginal Resection

Though brain metastases are often sharply demarcated on neuroimaging and grossly delimited by glial pseudo-capsules, histopathological studies have identified distinct invasion patterns of BMs,^51,52^ and the presence of infiltrating metastatic cells beyond the glial pseudo-capsule has been shown to significantly impact overall survival.^53^ Often determined by the surgeon’s subjective visual evaluation or a postoperative MRI, GTR may not detect microscopic infiltrating metastatic cells, which may partly account for the 46–57% of patients who experience local recurrence without subsequent radiotherapy.^54,55^ Supramarginal or microscopic total resection (MTR), in which the GTR margin is extended by 5 mm and the new margin is confirmed to be tumor-free by intraoperative frozen sectioning, has been studied as a technique to improve local control and progression of metastatic disease. In a retrospective study by Yoo et al.,^56^ 94 patients with a single brain metastasis underwent either MTR (*n* = 43) if the tumor was located in non-eloquent areas or GTR (*n* = 51) if the tumor was located in eloquent regions, followed by systemic chemotherapy with or without radiotherapy. The 2-year local recurrence rates (29.1% for MTR vs. 63.2% for GTR) and 2-year survival rates (27.3% for MTR and 3.8% for GTR) were significantly different, though median survival time between the two groups was not significantly different.^56^ In a retrospective study by Kamp et al.,^57^ complete supramarginal resection with electrophysiological monitoring was successfully implemented in 19 cases of eloquent BMs. No patients suffered new permanent neurologic deficits, and 15.7% experienced temporary deficits. Neurologic symptoms improved in five patients.^57^ In Pessina et al.,^58^ a retrospective study of 69 patients with single large (> 2.1 cm) BMs, supramarginal resection with adjuvant SRS enabled a 1- to 2-year local control of 100% and a median survival of 24 months. Of note, the association between supramarginal resection and increased median survival may be influenced by the study’s inclusion criteria of patients with controlled extracranial disease. Some studies have shown that greater extent of surgical resection prolongs survival only in cohorts with controlled extracranial disease.^59,60^ Overall, studies of supramarginal resection suggest that maximizing the extent of resection can improve the recurrence rates and survival of patients with BMs. These preliminary results could be further investigated with prospective and randomized studies.

### Brachytherapy

Brachytherapy involves implantation of radioactive isotopes into a tumor cavity and has been investigated as both a primary and adjuvant therapy for BMs. Two modalities have been primarily studied for the treatment of brain metastases: iodine-125 (^125^I) and cesium-131 (^131^Cs) brachytherapy.^61^ Though^125^I brachytherapy is the more widely studied and has been associated with local control and overall survival rates comparable to SRS.^62,63^ clinical adoption has been limited by high rates of radiation necrosis (up to 30%).^64^ High rates of radiation necrosis in ^125^I brachytherapy trials have been attributed to the long half-life of ^125^I and shrinkage of the tumor cavity shifting the position of radioactive seeds.^65^

Compared to ^125^I, ^131^Cs brachytherapy is a more promising modality with similarly high local control rates and a more favorable side effect profile. In a phase I/II study of surgical resection with^131^Cs brachytherapy for newly diagnosed brain metastases, Wernicke et al.^66^ demonstrated 100% local control, median overall survival of 9.9 months, and no cases of radiation necrosis. In a follow-up prospective study of surgical resection with^131^Cs brachytherapy for large BMs (≥ 2.0 cm), 100% local control was achieved, as well as a median overall survival of 15.1 months and no cases of radiation necrosis.^67^ Of note, 39% of the lesions were > 3.0 cm.^67^ The excellent local control rates of adjuvant^131^Cs brachytherapy for large BMs is significant given the comparatively poor local control rates of adjuvant SRS for large BMs (60.9% for BMs ≥ 3.0 cm vs. 92.5% for BMs < 3.0 cm).^68^ In a retrospective study, Wernicke et al. investigated surgical resection with^131^Cs brachytherapy as a salvage treatment for patients with recurrent brain metastases post-radiation (SRS and/or WBRT) and reported a 1-year actuarial local freedom from progression (FFP) of 83.3%, median overall survival of 7 months, and 1 case of asymptomatic radiation necrosis out of 15 patients.^69^ Though radiotherapy, such as SRS, is the definitive adjuvant for surgical resection, Julie et al. conducted a retrospective matched-pair analysis of surgery with adjuvant^131^Cs brachytherapy (*n* = 30) vs. surgery with adjuvant SRS (n = 60) and demonstrated a significantly lower local recurrence, significantly increased local-recurrence-free survival, and significantly increased distant-recurrence-free survival (DRFS) with^131^Cs brachytherapy. The local benefits of ^131^Cs brachytherapy were thought to reflect radiobiological advantages, improved action against larger tumors, and a shorter delay between resection and delivery of adjuvant therapy. The significantly improved DRFS was not anticipated and was speculated to be due to either delays in initiating or continuing systemic treatments for SRS patients or unaccounted differences between the two cohorts.^70^ Lastly, a prospective study by Pham *et al.* suggests that^131^Cs brachytherapy may support stable or improved functional recovery as measured by FACT-Br and MMSE scores.^71^ These results are favorable given that prior studies have associated radiotherapy with post-operative neurocognitive decline.^72,73^

The relative improvements in efficacy and safety, particularly the dramatic decrease in radiation necrosis, of ^131^Cs brachytherapy compared to ^125^I are related to differences in radiobiological properties and improved methodology in delivering the isotopes.^131^Cs has a shorter half-life, which limits radiation exposure to the patient, and a higher dose rate, which delivers a greater proportion of the dose in a shorter time.^66^ Furthermore, in the studies conducted by Wernicke et al.,^131^Cs was delivered at lower doses, with lower seed activity, and through a “seeds-on-a-string” technique that prevented cavity shrinkage, which increases the proximity of surrounding tissue and the risk of necrosis.^74^ In conclusion, adjuvant^131^Cs brachytherapy has demonstrated promising benefits, especially with larger lesions, as well as significantly lower rates of radiation necrosis than^125^I brachytherapy. A randomized controlled trial comparing post-surgical^131^Cs brachytherapy vs. SRS for newly diagnosed brain metastases is currently underway and will provide crucial data on the appropriate use-cases of brachytherapy (NCT 04365374, Clinicaltrials.gov).

## Intraoperative Augmentation

### Neuronavigation

First introduced by Roberts et al. in 1986,^75^ frameless stereotactic navigation systems, or neuronavigation, have become an essential intraoperative tool in predicting the location of target lesions and neighboring regions at risk during surgery. Neuronavigation is established through four steps.^76^ First, preoperative images are acquired. MRI allows high-resolution visualization of the parenchyma and soft tissue, while fMRI and DTI localize eloquent cortex and white matter fiber tracks. Second, the images are uploaded into the neuronavigation system. Third, the images are registered or mapped onto the physical patient *via* homologous landmark/fiducial matching or surface matching. Lastly, a tracked tool in the physical space allows identification of the corresponding anatomy in the images, typically displayed on a monitor in the operating room (OR). Patient-to-image registration has a paired point matching accuracy between 1.6 and 6.2 mm.^76–78^

In clinical practice, neuronavigation is ubiquitous, facilitating the surgical resection of brain metastases.^79^ In Schackert et al.,^80^ neuronavigation prolonged median survival time in patients with single (16 months vs. 10 months) and multiple (11 months vs. 5 months) metastases, though these results were not statistically significant. The lack of statistical significance was partially explained by brain shift and a biased selection criteria for neuronavigation, which was used for patients with deep-seated or near-eloquent lesions and not for patients with uncomplicated lesions. In Tan and Black, image-guided resection of BMs decreased length of hospital stay, improved functional status, and prolonged survival.^81^ A primary limitation of conventional neuronavigational systems is a lack of accommodation for brain shift—movements in the patient’s anatomy during surgery from parenchymal swelling, resection, gravity, CSF drainage, and other factors.^76^ Lesion size affects the degree of brain shift with smaller tumors (< 30 cm^3^) not significantly altering the success of neuronavigation-guided resection.^82^ Intraoperative imaging, which is discussed in a subsequent section, may facilitate more accurate real-time visualization of a shifting brain parenchyma.

### Intraoperative Ultrasound

First applied to adult neurosurgery in the 1980s,^83^ intraoperative ultrasound (iUS) has become an essential neurosurgical tool. Current modalities include 2D US, 3D US, contrast-enhanced US (ceUS), high-frequency US (hfUS), and US elastography.^84^ In general, iUS has been studied for three main applications: intraoperative navigation, assessing the extent of resection, and measuring/compensating for brain shift.^84^ The benefits of iUS include its low cost, minimal disruption of intraoperative workflows, and a lack of radiation exposure. While brain parenchyma is largely uniform in echogenicity on iUS with gray matter slightly more hyperechoic than white matter, tumors are relatively hyperechoic due to high mass density.^84^

2D US is the most basic modality that has been applied to BMs. In Di Lorenzo et al.,^85^ 2D iUS-guided tumor biopsies were faster and less costly than CT-guided biopsies and yielded comparable rates of histological diagnosis on first biopsy procedure. In a prospective study by Hammoud et al.,^86^ 2D iUS reliably localized 100% of 34 BMs and accurately determined the extent of resection in all cases, as confirmed by a mean difference of zero between postexcision tumor volumes measured by iUS and postoperative MRI. However, iUS was less precise for recurrent tumors with prior surgery or radiation, possibly due to postintervention changes complicating the tissue echogenicity.^86^ In a case series including three patients with BMs, LeRoux *et al*. showed that 2D iUS improved identification of tumor margins compared to both contrast and non-contrast T1-weighted MRI and helped distinguish tumor and normal brain from edema visualized on T2-weighted MRI.^87^ In a retrospective series by Serra et al.,^88^ hfUS facilitated GTR in all 8 BM cases. iUS can improve not only the localization and resection of BMs, but also postoperative performance. In a retrospective study by de Lima Oliveira et al.,^89^ 78 BM patients underwent surgery either with iUS (*n* = 35) or without (*n* = 43) iUS. Compared to the control group, the iUS group had significantly higher postoperative KPS scores and a significantly higher proportion of patients who improved their KPS scores, especially with moderately difficult tumor resections, eloquent tumors, tumors not associated with vessels or nerve, and solitary lesions.^89^ Furthermore, the residual tumor volume, as assessed by postoperative MRI, was significantly lower in the iUS group than in the control group.^89^

ceUS utilizes contrast agents containing microparticles and air bubbles to enable enhanced real-time visualization of the cerebral vasculature.^90^ In Kanno et al.,^90^ the first study to evaluate ceUS in tumor resections, most metastatic tumors demonstrated strong signals on ceUS. Moreover, there was a more significant correlation between ceUS and digital subtraction (DS) angiograms than between ceUS and contrast-enhanced CT and MRI, highlighting that US contrast enhancement is more closely related to vascularity while CT or MR contrast enhancement is related to destruction of the blood-brain barrier.^90^ As such, ceUS can aid BM resection by identifying feeding arteries, draining veins, and tumor vasculature and by confirming total resection through the disappearance of Doppler signal.^90^ The utility of intraoperative ceUS was confirmed in Engelhardt et al.,^91^ a prospective study in which metastatic visualization was enhanced with contrast and hyperechoic tumors were distinguished from hypoechoic peritumoral edema. Similarly, in Prada et al.,^92^ all brain metastases appeared markedly hyperechoic on ceUS, and contrast enhancement clearly defined the tumor borders.

US elastography measures the elastic properties of tissue either through controlled low-frequency vibrations generated by the probe (vibrography) or through observations of arterial pulsations.^93,94^ In Scholz et al.,^93^ vibrography measurements of metastatic carcinoma demonstrated a range of strain: lower strain than brain tissue, identical strain to brain tissue, or inhomogeneous strain. In Chauvet et al.,^95^ intraoperative shear wave elastography significantly differentiated metastases from meningiomas based on elasticity. In Prada et al.,^96^ intraoperative strain elastography (SE) based on brain pulsatility measured BMs as either stiffer (kidney, colon) or softer (endometrial, lung) than normal brain. In 64% of the study’s 64 cases, including gliomas, meningiomas, metastases, and other tumor types, SE differentiated lesion margins more sharply than 2D B-mode US.

3D iUS reconstructs 2D slices into volumetric images that may be reviewed in any plane and can accurately pinpoint tumors and vessels in a more intuitive way than 2D techniques.^97,98^ In Unsgaard et al.,^99^ there was 100% concordance between 3D iUS and histopathologic biopsy in differentiating BMs from non-BM tissue, while T1- and T2-weighted MRI yielded 90% and 86% concordance with histopathology, respectively. In Rygh et al,^100^ 3D-iUS angiography with power Doppler was a useful intraoperative neuronavigational tool, enabling identification of hidden vessels in 3 out of 5 BM cases and therefore improving spatial perception of risk structures. Of note, power Doppler is less angle-dependent than color flow Doppler, allowing better reconstruction of vessel continuity.^100^ In Tronnier et al.,^101^ 3D-navigated US offered a primary visualization of metastases that was comparable to intraoperative MRI.

iUS modalities are being combined with preexisting imaging techniques, such as MRI-based neuronavigation,^102^ MRI tractography,^103^ and augmented reality,^104^ to improve real-time visualization of the brain parenchyma and to monitor and compensate for brain shift. Most of these applications depend on an algorithmic registration process that enables parenchymal shifts detected intraoperatively by iUS to update preoperative MRI images.^84^ Registration is technically challenging since US and MR images highlight different anatomic details and generate unique artifacts and noise patterns.^105^ Development of brain-shift-monitoring technologies is still in a relatively early stage, but preliminary results are promising.^105^

### Intraoperative Brain Mapping

Complete resection of brain metastases in patients with well-controlled systemic disease can improve survival.^59,60^ Tumors located in eloquent brain regions are particularly challenging to resect completely. Preoperative imaging, such as fMRI, DTI, and TMS, and intraoperative neuronavigation can aid surgical planning, but these techniques are susceptible to brain shift and limited in resolution. Intraoperative brain mapping refers to neurophysiological methods that precisely identify brain areas associated with motor, sensory, language, and other neurological functions and therefore enable maximal resection of eloquent lesions.

Direct cortical and subcortical stimulation has been the gold standard for intraoperative brain mapping since the 1930s.^106^ The motor cortex can be identified by stimulating the pre and postcentral gyri, premotor area, and supplementary motor area and either observing the extremities for contralateral movement or measuring action potentials recorded by peripheral electrodes, also referred to as motor-evoked potentials (MEPs).^107^ Alternatively, the central sulcus, primary motor cortex, and primary somatosensory cortex can be identified by stimulating the contralateral median, ulnar, or posterior tibial nerves and measuring sensory evoked potentials (SEPs) on the cortical surface.^108^ The central sulcus is the location of “phase reversal.” ^108^ Subcortical structures, including white matter, can also be stimulated and assessed with MEPs or other functional readouts.^109^ Awake craniotomies, in which electrical stimulation is applied to cortical or subcortical structures while patients perform intraoperative tasks, can also identify brain regions involved in language, calculation, motor, somatosensory, and visual functions.^110^ Though brain mapping has been more extensively studied in gliomas,^111–113^ these techniques have been safely and effectively applied to BMs.

Intraoperative neurophysiological monitoring with MEPs has facilitated safe, efficacious, and neurologically beneficial resection of BMs. In a retrospective study by Krieg et al.,^114^ 56 eloquent metastases were resected with intraoperative MEPs monitoring. A threshold of > 80% reduction in MEP amplitude yielded a lower false-positive rate and correlated better with postoperative outcomes than > 50% reduction,^114^ which has been previously recommended for glioma surgery.^115^ Surgery improved the strength of 21% of patients.^114^ New permanent motor deficits (12.5% of cases) were associated with location of the tumor (precentral gyrus > corticospinal tract > insula), preoperative motor deficits, preoperative radiotherapy, and recursive partitioning analysis (RPA) class 3.^114^ In another retrospective study including 56 BMs, Obermueller *et al.,* confirmed that > 80% reduction in MEP amplitude was a more appropriate BM-specific alarm threshold than > 50% reduction.^116^ In this cohort, compared to gliomas, the BM group had more stable MEPS, a significantly higher proportion of patients with improved postoperative neurological status, and less subtotal resection.^116^ These two studies support the use of intraoperative MEP monitoring in the resection of motor-eloquent BMs.

Awake craniotomies for BMs have also been studied. In Chua et al.,^117^ data from 7 studies and 104 patients with eloquent BMs who underwent awake craniotomies were aggregated. Gross total or supramarginal resection was achieved in 93% of patients,^117^ which is higher than the mean GTR of glioma patients who underwent intraoperative mapping.^113^ The overall local recurrence rate of 3 studies (70 patients) was 9%, and the median survival of 2 studies (36 patients) was 12–16.2 months.^117^ These findings are comparable to historical rates in the literature.^54^ Furthermore, 73% of patients had no change or improvement in neurologic outcomes, and only one out of 104 patients developed late neurologic deficits after awake craniotomies.^117^ Thus, awake craniotomies can safely and effectively treat BMs.

Intraoperative brain mapping has been combined with other surgical innovations to optimize the resection of BMs. In a prospective study by Krieg et al., 250 patients with peri-Rolandic metastatic lesions were preoperatively evaluated with (*n* = 120) or without (*n* = 130) navigated TMS (nTMS). The nTMS group had lower rates of intraoperative MEPs monitoring, shorter surgical times, lower rates of residual tumor, decreased surgery-related paresis, and smaller craniotomies. These results demonstrate how preoperative TMS can improve the implementation of intraoperative brain mapping and ensure that the technique is only used for cases in which functional regions are truly at-risk.^118^ Lastly, intraoperative neurophysiological monitoring has been augmented with 3D US to optimize the resection of BMs.^119,120^

### Endoscope

First developed by Dandy to visualize and treat intraventricular pathologies, the endoscope has become an essential intraoperative tool for facilitating minimally invasive approaches and for resecting intraventricular, paraventricular, sellar, pineal, and other deep-seated lesions.^121^ Though initially limited by poor lighting and magnification, advances in lens development, fiberoptics, and device design have enabled high-definition, 3D, angled visualization.^121^ Compared to OMs, endoscopes provide wider fields of view in small spaces and are typically paired with less invasive approaches.^27^ Limitations include a constrained surgical working space, short focal/field depths, and technical difficulties with larger lesions.^121^ When applied to tumor surgery, an endoscope can serve either as the primary mode of visualization, or as an adjunct to access views not possible with the OM and to evaluate the extent of resection.^27^

Several studies have evaluated the endoscopic resection of BMs located in various intracranial regions. In a prospective series by Plaha et al.,^122^ endoscope-assisted bimanual microsurgery achieved total resection in 92% of 12 metastases located in the temporal and frontal lobes, parafalcine region, cerebellum, and other areas. Ma et al.^123^ applied endoscope-assisted bimanual microsurgery to temporal lobe metastases and reported GTR in 63.6%, a median overall survival of 12.9 months, and shorter hospital stays. Barkhoudarian et al.^124^ developed an endoscope-assisted transfalcine approach for eleven contralateral deep medial cortical metastases and demonstrated how endoscopes can visualize residual tumor missed by OMs, allowing additional resection in 91% of cases. In a retrospective study by Zacharia et al.,^125^ endoscopic endonasal resection of twelve anterior skull base metastases led to an overall survival of 16 months and median progression-free survival of 18 months. These survival outcomes were better than prior studies of skull base metastases.^125^ Endoscopes have also supplemented other minimally invasive approaches, such as the SO craniotomy, enabling more direct illumination in deep operative fields and visualization of regions previously hidden from view.^126^ Ports or tubular retractors can further facilitate endoscopic resection of intraparenchymal, deep-seated, and intraventricular metastases.^127^ Ports are typically large enough to accommodate a rigid endoscope and at least two instruments, allowing bimanual microsurgical dissection.^127^ Despite these benefits, a retrospective study by Hong *et al.* suggested that endoscopes may be more useful as an adjunct for inspection of the tumor bed, rather than a primary visualization tool.^45^ Lastly, devices designed for narrow surgical corridors, such as a side-cutting variable aspiration instrument (NICO Myriad, Indianapolis, IN), can augment the safety and efficacy of endoscopic BM resection.^128^

### Exoscope

Exoscopes are telescope-based video systems with long optical working distances and wide fields of view that provide high-definition 2D or 3D views of the surgical field. Developed over the past decade, the exoscope combines the manageability and high-definition monitor-based views of endoscopes and the magnification, lighting, stereopsis, and dissection quality of OMs.^129^ In a systematic review of 29 studies and 574 patients, Ricciardi et al. showed that exoscopes are superior or equivalent to OMs in ergonomic comfort, image quality, magnification, lighting, costs, quality of microsurgical dissection, and educational opportunities for surgeons, trainees, and OR staff.^129^ Limitations include a lack of stereopsis in 2D exoscopes, though this has been resolved with 3D models, physical discomfort from prolonged usage of 3D glasses, and difficult repositioning, though this has also been addressed with models that include a foot-pedal controller and a sterile pilot unit.^129^ Current exoscopes include the VITOM 2D or 3D and HDXO-scope, BrightMatter, ORBEYE, and KINEVO 900.

First implemented by Mamelak et al.,^130^ exoscopes have been applied to the surgical management of BMs. In Roethe et al., an exoscopic visualization system was used to resect 3 cases of metastatic lesions out of a total 20 randomized supratentorial tumors. The study found that frontoparietal and pterional approaches were better suited for exoscopic visualization than retrosigmoid or suboccipital approaches. Furthermore, there were limitations in visualizing deep lesions and small bleeding vessels, and participants concluded that the exoscope was not sufficient to replace the OM.^131^ In Oertel and Burkhardt,^132^ the VITOM-3D exoscope enabled the resection of three frontal or temporal metastatic lesions, though the authors also reported limitations with deep-seated tumors and tissue identification during bleeding. When paired with other surgical innovations, such as tubular retractors, the exoscope can enable the resection of more complex lesions. For example, studies have reported 64–95% GTR rates, stable or improved postoperative neurological function, and shorter hospital stays for subcortical and posterior fossa BMs using exoscopes and tubular retractors.^41,46,47^ In Marenco-Hillembrand *et al*., exoscopic visualization of burr hole-based resections achieved GTR and stable or improved KPS scores in all 8 BM cases. This study proved that exoscopes can be used at the extreme limit of keyhole surgeries without compromising surgical outcomes.^133^ Lastly, exoscopes have enabled new intraoperative fluorescent imaging techniques, such as second-window indocyanine green (SWIG), discussed in the subsequent section.^134,135^ In conclusion, several reports have demonstrated safe and effective exoscope-based resection of metastatic lesions.

### Fluorescence-Guided Surgery

Since the 1940s,^136^ fluorescent dyes have been studied to improve intraoperative delineation of tumor from normal parenchyma and optimize the extent of resection. 5-aminolevulinic acid (5-ALA), a precursor of the heme synthesis pathway that is converted to fluorescent protoporphyrin IX (PpIX), has been shown to significantly increase the rate of complete resection and progression-free survival in gliomas,^137^ yet similar benefits have not been found with BMs.^138–140^ Two other FDA-approved fluorophores—fluorescein and indocyanine green (ICG)—have demonstrated more promising results for BMs.

Applied to brain tumor surgery since the 1940s,^136^ fluorescein is a yellow–green xanthine fluorophore that passively extravagates into brain tissues with disrupted blood–brain barrier. Most studies employ a YELLOW 560 nm filter directly integrated into the OM to visualize the fluorescent signal. With fluorescein labeling, 90–100% of BMs demonstrate strong signal,^141–144^ though visualization is limited in cases of pigmented melanoma, hemorrhagic metastases, or previously irradiated tissue.^145^ Across several retrospective cohorts, fluorescein visualization facilitated GTR in 83.3–100% of cases.^142,143,146^ Retrospective studies comparing fluorescein-guided surgery and white light-guided surgery suggest that fluorescein may be associated with better GTR rates, postoperative KPS, and survival.^144,147^ In conclusion, fluorescein can safely and consistently localize BMs, though its clinical value is yet to be definitively determined.

Indocyanine green (ICG) is a hydrophobic cyanine dye that binds to intravascular plasma proteins and is visualized with near-infrared (NIR) cameras.^148^ Since NIR light has a longer wavelength than visible light, signal from the tumor can be viewed through the dura and up to 20 mm through normal parenchyma, which can facilitate precise dural incisions and neuronavigation unaffected by brain shift.^134^ Recently, Lee and colleagues developed a technique called second window indocyanine green (SWIG), in which a high dose of ICG is administered 24 hours prior to surgery and accumulates in tumor tissue *via* increased permeability and retention effect.^149^ In a prospective study by Lee et al., the sensitivity, specificity, positive predictive value, and negative predictive value of SWIG was 96.4%, 27.3%, 77.1%, and 75.0%, respectively, compared to 82.1%, 90.9%, 95.8%, and 66.7% for white light; therefore, SWIG improved the sensitivity of tumor detection at the expense of specificity. GTR was achieved in 77% of 13 BMs and in 100% of lesions not previously treated.^150^ In another prospective study by Muto et al.^134^ SWIG enabled GTR in 90% of 10 BMs with complete resection corresponding with an absence of NIR signal. Lastly, in a prospective study by Teng et al.,^135^ compared to postoperative MRI, absence of NIR signal was a better predictor of GTR, reduced recurrence rate, and improved progression-free survival. In conclusion, SWIG-guided surgery is a promising approach for the treatment of BMs still under investigation..

## Conclusion

From sophisticated preoperative imaging to new techniques in operative approach and intraoperative augmentation, neurosurgical advances have dramatically altered the treatment of patients with brain metastases, the most common intracranial adult tumor. Keyhole craniotomies and tubular retractors represent a movement toward more minimally invasive neurosurgical approaches, ensuring that patients receive optimal care while minimizing morbidity. Supramarginal surgery has pushed the boundaries of achieving extent of resection. Brachytherapy has highlighted the potential of locally delivering therapeutic agents to the resection cavity. Innovations in neuronavigation, iUS, brain mapping, endoscopes, exoscopes, and fluorescent stains have enabled increasingly effective high-definition, real-time visualizations of the brain. Pushed forward by these multidisciplinary innovations, neurosurgery has never been a safer, more effective treatment for patients with brain metastases.

## References

[1] Ostrom QT , Wright CH, Barnholtz-Sloan JS. Brain metastases: epidemiology. Handb Clin Neurol. 2018;149:27–42. 29307358 10.1016/B978-0-12-811161-1.00002-5

[2] Schouten LJ , Rutten J, Huveneers HA, Twijnstra A. Incidence of brain metastases in a cohort of patients with carcinoma of the breast, colon, kidney, and lung and melanoma. Cancer. 2002;94(10):2698–2705. 12173339 10.1002/cncr.10541

[3] Barnholtz-Sloan JS , Sloan AE, Davis FG, Vigneau FD, Lai P, Sawaya RE. Incidence proportions of brain metastases in patients diagnosed (1973 to 2001) in the Metropolitan Detroit Cancer Surveillance System. J Clin Oncol. 2004;22(14):2865–2872. 15254054 10.1200/JCO.2004.12.149

[4] Posner JB , Chernik NL. Intracranial metastases from systemic cancer. Adv Neurol. 1978;19:579–592. 570349

[5] Takakura K , ed. Metastatic Tumors of the Central Nervous System. 1st ed. Tokyo, Japan: Igaku-Shoin; 1982.

[6] Fox BD , Cheung VJ, Patel AJ, Suki D, Rao G. Epidemiology of metastatic brain tumors. Neurosurg Clin N Am. 2011;22(1):1–6, v. 21109143 10.1016/j.nec.2010.08.007

[7] Bindal RK , Sawaya R, Leavens ME, Hess KR, Taylor SH. Reoperation for recurrent metastatic brain tumors. J Neurosurg. 1995;83(4):600–604. 7674007 10.3171/jns.1995.83.4.0600

[8] Bindal AK , Bindal RK, Hess KR, et al. Surgery versus radiosurgery in the treatment of brain metastasis. J Neurosurg. 1996;84(5):748–754. 8622147 10.3171/jns.1996.84.5.0748

[9] Pope WB . Brain metastases: neuroimaging. Handb Clin Neurol. 2018;149:89–112. 29307364 10.1016/B978-0-12-811161-1.00007-4 PMC6118134

[10] Sze G , Milano E, Johnson C, Heier L. Detection of brain metastases: comparison of contrast-enhanced MR with unenhanced MR and enhanced CT. AJNR Am J Neuroradiol. 1990;11(4):785–791. 2114769 PMC8331625

[11] Schellinger PD , Meinck HM, Thron A. Diagnostic accuracy of MRI compared to CCT in patients with brain metastases. J Neurooncol. 1999;44(3):275–281. 10720207 10.1023/a:1006308808769

[12] Luna LP , Sherbaf FG, Sair HI, Mukherjee D, Oliveira IB, Köhler CA. Can preoperative mapping with functional MRI reduce morbidity in brain tumor resection? A systematic review and meta-analysis of 68 observational studies. Radiology. 2021;300(2):338–349. 34060940 10.1148/radiol.2021204723

[13] Raffa G , Scibilia A, Conti A, et al. The role of navigated transcranial magnetic stimulation for surgery of motor-eloquent brain tumors: a systematic review and meta-analysis. Clin Neurol Neurosurg. 2019;180:7–17. 30870762 10.1016/j.clineuro.2019.03.003

[14] Potgieser AR , Wagemakers M, van Hulzen AL, de Jong BM, Hoving EW, Groen RJ. The role of diffusion tensor imaging in brain tumor surgery: a review of the literature. Clin Neurol Neurosurg. 2014;124:51–58. 25016239 10.1016/j.clineuro.2014.06.009

[15] National Comprehensive Cancer Network. NCCN Guidelines. Published online 2021. https://www.nccn.org/guidelines/category_1. Accessed June 1, 2021.

[16] Achrol AS , Rennert RC, Anders C, et al. Brain metastases. Nat Rev Dis Primers. 2019;5(1):5. 30655533 10.1038/s41572-018-0055-y

[17] Berghoff AS , Bartsch R, Wöhrer A, et al. Predictive molecular markers in metastases to the central nervous system: recent advances and future avenues. Acta Neuropathol. 2014;128(6):879–891. 25287912 10.1007/s00401-014-1350-7

[18] Brastianos PK , Carter SL, Santagata S, et al. Genomic characterization of brain metastases reveals branched evolution and potential therapeutic targets. Cancer Discov. 2015;5(11):1164–1177. 26410082 10.1158/2159-8290.CD-15-0369 PMC4916970

[19] Ewend MG , Morris DE, Carey LA, Ladha AM, Brem S. Guidelines for the initial management of metastatic brain tumors: role of surgery, radiosurgery, and radiation therapy. J Natl Compr Canc Netw. 2008;6(5):505–13; quiz 514. 18492462 10.6004/jnccn.2008.0038

[20] Patchell RA , Tibbs PA, Walsh JW, et al. A randomized trial of surgery in the treatment of single metastases to the brain. N Engl J Med. 1990;322(8):494–500. 2405271 10.1056/NEJM199002223220802

[21] Vecht CJ , Haaxma-Reiche H, Noordijk EM, et al. Treatment of single brain metastasis: radiotherapy alone or combined with neurosurgery? Ann Neurol. 1993;33(6):583–590. 8498838 10.1002/ana.410330605

[22] Bindal RK , Sawaya R, Leavens ME, Lee JJ. Surgical treatment of multiple brain metastases. J Neurosurg. 1993;79(2):210–216. 8331402 10.3171/jns.1993.79.2.0210

[23] Paek SH , Audu PB, Sperling MR, Cho J, Andrews DW. Reevaluation of surgery for the treatment of brain metastases: review of 208 patients with single or multiple brain metastases treated at one institution with modern neurosurgical techniques. Neurosurgery. 2005;56(5):1021–34; discussion 1021. 15854250

[24] Salvati M , Tropeano MP, Maiola V, et al. Multiple brain metastases: a surgical series and neurosurgical perspective. Neurol Sci. 2018;39(4):671–677. 29383618 10.1007/s10072-017-3220-2

[25] Nahed BV , Alvarez-Breckenridge C, Brastianos PK, et al. Congress of Neurological Surgeons Systematic Review and Evidence-Based Guidelines on the role of surgery in the management of adults with metastatic brain tumors. Neurosurgery. 2019;84(3):E152–E155. 30629227 10.1093/neuros/nyy542

[26] Perneczky A , Reisch R. Keyhole Approaches in Neurosurgery: Volume I Concept and Surgical Technique. Vienna, Austria: Springer-Verlag Wien; 2008.

[27] Garrett M , Consiglieri G, Nakaji P. Transcranial minimally invasive neurosurgery for tumors. Neurosurg Clin N Am. 2010;21(4):595–605, v. 20947029 10.1016/j.nec.2010.07.002

[28] Teo C. Principles and Practice of Keyhole Brain Surgery. Stuttgart, Germany: Georg Thieme Verlag; 2019.

[29] Reisch R , Stadie A, Kockro RA, Hopf N. The keyhole concept in neurosurgery. World Neurosurg. 2013;79(2 Suppl):S17.e9–S17.13. 10.1016/j.wneu.2012.02.024 22381839

[30] Tobler WD , Stanley M. Stereotactic resection of brain metastases in eloquent brain. Stereotact Funct Neurosurg. 1994;63(1-4):38–44. 7624649 10.1159/000100289

[31] Phang I , Leach J, Leggate JRS, Karabatsou K, Coope D, D’Urso PI. Minimally invasive resection of brain metastases. World Neurosurg. 2019;130:e362–e367. 31233927 10.1016/j.wneu.2019.06.091

[32] Baker CM , Glenn CA, Briggs RG, et al. Simultaneous resection of multiple metastatic brain tumors with multiple keyhole craniotomies. World Neurosurg. 2017;106:359–367. 28652117 10.1016/j.wneu.2017.06.118

[33] Ditzel Filho LF , McLaughlin N, Bresson D, Solari D, Kassam AB, Kelly DF. Supraorbital eyebrow craniotomy for removal of intraaxial frontal brain tumors: a technical note. World Neurosurg. 2014;81(2):348–356. 23352966 10.1016/j.wneu.2012.11.051

[34] Reisch R , Perneczky A, Filippi R. Surgical technique of the supraorbital key-hole craniotomy. Surg Neurol. 2003;59(3):223–227. 12681560 10.1016/s0090-3019(02)01037-6

[35] Reisch R , Marcus HJ, Hugelshofer M, Koechlin NO, Stadie A, Kockro RA. Patients’ cosmetic satisfaction, pain, and functional outcomes after supraorbital craniotomy through an eyebrow incision. J Neurosurg. 2014;121(3):730–734. 24878288 10.3171/2014.4.JNS13787

[36] Eroglu U , Shah K, Bozkurt M, et al Supraorbital keyhole approach: lessons learned from 106 operative cases. World Neurosurg. 2019;124:e667–e674. 10.1016/j.wneu.2018.12.188 30659969

[37] Bonney PA , Boettcher LB, Cheema AA, Maurer AJ, Sughrue ME. Operative results of keyhole supracerebellar-infratentorial approach to the pineal region. J Clin Neurosci. 2015;22(7):1105–1110. 25913279 10.1016/j.jocn.2014.12.029

[38] Andrews RJ , Bringas JR. A review of brain retraction and recommendations for minimizing intraoperative brain injury. Neurosurgery. 1993;33(6):1052–63; discussion 1063. 8133991 10.1227/00006123-199312000-00014

[39] Kelly PJ , Goerss SJ, Kall BA. The stereotaxic retractor in computer-assisted stereotaxic microsurgery. Technical note. J Neurosurg. 1988;69(2):301–306. 3292721 10.3171/jns.1988.69.2.0301

[40] Greenfield JP , Cobb WS, Tsouris AJ, Schwartz TH. Stereotactic minimally invasive tubular retractor system for deep brain lesions. Neurosurgery. 2008;63(4 Suppl 2):334–9; discussion 339. 18981840 10.1227/01.neu.0000334741.61745.72

[41] Day JD . Transsulcal parafascicular surgery using Brain Path® for subcortical lesions. Neurosurgery. 2017;64(CN_suppl_1):151–156. 28899061 10.1093/neuros/nyx324

[42] Bander ED , Jones SH, Kovanlikaya I, Schwartz TH. Utility of tubular retractors to minimize surgical brain injury in the removal of deep intraparenchymal lesions: a quantitative analysis of FLAIR hyperintensity and apparent diffusion coefficient maps. J Neurosurg. 2016;124(4):1053–1060. 26430838 10.3171/2015.4.JNS142576

[43] Echeverry N , Mansour S, MacKinnon G, Jaraki J, Shapiro S, Snelling B. Intracranial tubular retractor systems: a comparison and review of the literature of the BrainPath, Vycor, and METRx tubular retractors in the management of deep brain lesions. World Neurosurg. 2020;143:134–146. 32717353 10.1016/j.wneu.2020.07.131

[44] Eichberg DG , Di L, Shah AH, et al. Minimally invasive resection of intracranial lesions using tubular retractors: a large, multi-surgeon, multi-institutional series. J Neurooncol. 2020;149(1):35–44. 32556805 10.1007/s11060-020-03500-0

[45] Hong CS , Prevedello DM, Elder JB. Comparison of endoscope- versus microscope-assisted resection of deep-seated intracranial lesions using a minimally invasive port retractor system. J Neurosurg. 2016;124(3):799–810. 26315005 10.3171/2015.1.JNS141113

[46] Bakhsheshian J , Strickland BA, Jackson C, et al. multicenter investigation of channel-based Subcortical trans-Sulcal Exoscopic resection of metastatic brain tumors: a retrospective case series. Oper Neurosurg (Hagerstown). 2019;16(2):159–166. 29912398 10.1093/ons/opy079

[47] Mampre D , Bechtle A, Chaichana KL. Minimally invasive resection of intra-axial posterior Fossa tumors using tubular retractors. World Neurosurg. 2018;119:e1016–e1020. 30130571 10.1016/j.wneu.2018.08.049

[48] Akbari SHA , Sylvester PT, Kulwin C, et al. Initial experience using intraoperative magnetic resonance imaging during a trans-sulcal tubular retractor approach for the resection of deep-seated brain tumors: a case series. Oper Neurosurg (Hagerstown). 2019;16(3):292–301. 29850853 10.1093/ons/opy108 PMC6506631

[49] Zammar SG , Cappelli J, Zacharia BE. Utility of tubular retractors augmented with Intraoperative ultrasound in the resection of deep-seated brain lesions: technical note. Cureus. 2019;11(3):e4272. 31157134 10.7759/cureus.4272 PMC6529054

[50] Bander ED , Jones SH, Pisapia D, et al. Tubular brain tumor biopsy improves diagnostic yield for subcortical lesions. J Neurooncol. 2019;141(1):121–129. 30446900 10.1007/s11060-018-03014-w

[51] Berghoff AS , Rajky O, Winkler F, et al. Invasion patterns in brain metastases of solid cancers. Neuro Oncol. 2013;15(12):1664–1672. 24084410 10.1093/neuonc/not112 PMC3829586

[52] Baumert BG , Rutten I, Dehing-Oberije C, et al. A pathology-based substrate for target definition in radiosurgery of brain metastases. Int J Radiat Oncol Biol Phys. 2006;66(1):187–194. 16814946 10.1016/j.ijrobp.2006.03.050

[53] Siam L , Bleckmann A, Chaung HN, et al. The metastatic infiltration at the metastasis/brain parenchyma-interface is very heterogeneous and has a significant impact on survival in a prospective study. Oncotarget. 2015;6(30):29254–29267. 26299612 10.18632/oncotarget.4201 PMC4745724

[54] Patchell RA , Tibbs PA, Regine WF, et al. Postoperative radiotherapy in the treatment of single metastases to the brain: a randomized trial. JAMA. 1998;280(17):1485–1489. 9809728 10.1001/jama.280.17.1485

[55] Mahajan A , Ahmed S, McAleer MF, et al. Post-operative stereotactic radiosurgery versus observation for completely resected brain metastases: a single-centre, randomised, controlled, phase 3 trial. Lancet Oncol. 2017;18(8):1040–1048. 28687375 10.1016/S1470-2045(17)30414-X PMC5560102

[56] Yoo H , Kim YZ, Nam BH, et al. Reduced local recurrence of a single brain metastasis through microscopic total resection. J Neurosurg. 2009;110(4):730–736. 19072310 10.3171/2008.8.JNS08448

[57] Kamp MA , Dibué M, Niemann L, et al. Proof of principle: supramarginal resection of cerebral metastases in eloquent brain areas. Acta Neurochir (Wien). 2012;154(11):1981–1986. 22875595 10.1007/s00701-012-1463-5

[58] Pessina F , Navarria P, Cozzi L, et al. Role of surgical resection in patients with single large brain metastases: feasibility, morbidity, and local control evaluation. World Neurosurg. 2016;94:6–12. 27373936 10.1016/j.wneu.2016.06.098

[59] Agboola O , Benoit B, Cross P, et al. Prognostic factors derived from recursive partition analysis (RPA) of Radiation Therapy Oncology Group (RTOG) brain metastases trials applied to surgically resected and irradiated brain metastatic cases. Int J Radiat Oncol Biol Phys. 1998;42(1):155–159. 9747833 10.1016/s0360-3016(98)00198-9

[60] Lee CH , Kim DG, Kim JW, et al. The role of surgical resection in the management of brain metastasis: a 17-year longitudinal study. Acta Neurochir (Wien). 2013;155(3):389–397. 23325516 10.1007/s00701-013-1619-y

[61] Chitti B , Goyal S, Sherman JH, et al. The role of brachytherapy in the management of brain metastases: a systematic review. J Contemp Brachytherapy. 2020;12(1):67–83. 32190073 10.5114/jcb.2020.93543 PMC7073344

[62] Ruge MI , Kocher M, Maarouf M, et al. Comparison of stereotactic brachytherapy (125 iodine seeds) with stereotactic radiosurgery (LINAC) for the treatment of singular cerebral metastases. Strahlenther Onkol. 2011;187(1):7–14. 21234527 10.1007/s00066-010-2168-4

[63] Rogers LR , Rock JP, Sills AK, et al.; Brain Metastasis Study Group. Results of a phase II trial of the GliaSite radiation therapy system for the treatment of newly diagnosed, resected single brain metastases. J Neurosurg. 2006;105(3):375–384. 16961129 10.3171/jns.2006.105.3.375

[64] Bernstein M , Cabantog A, Laperriere N, Leung P, Thomason C. Brachytherapy for recurrent single brain metastasis. Can J Neurol Sci. 1995;22(1):13–16. 7750066 10.1017/s0317167100040439

[65] Mahase SS , Navrazhina K, Schwartz TH, Parashar B, Wernicke AG. Intraoperative brachytherapy for resected brain metastases. Brachytherapy. 2019;18(3):258–270. 30850332 10.1016/j.brachy.2019.01.011

[66] Wernicke AG , Yondorf MZ, Peng L, et al. Phase I/II study of resection and intraoperative cesium-131 radioisotope brachytherapy in patients with newly diagnosed brain metastases. J Neurosurg. 2014;121(2):338–348. 24785322 10.3171/2014.3.JNS131140 PMC4249933

[67] Wernicke AG , Hirschfeld CB, Smith AW, et al. Clinical Outcomes of Large Brain Metastases Treated With Neurosurgical Resection and Intraoperative Cesium-131 Brachytherapy: Results of a Prospective Trial. Int J Radiat Oncol Biol Phys. 2017;98(5):1059–1068. 28721889 10.1016/j.ijrobp.2017.03.044

[68] Brennan C , Yang TJ, Hilden P, et al. A phase 2 trial of stereotactic radiosurgery boost after surgical resection for brain metastases. Int J Radiat Oncol Biol Phys. 2014;88(1):130–136. 24331659 10.1016/j.ijrobp.2013.09.051 PMC5736310

[69] Wernicke AG , Smith AW, Taube S, et al. Cesium-131 brachytherapy for recurrent brain metastases: durable salvage treatment for previously irradiated metastatic disease. J Neurosurg. 2017;126(4):1212–1219. 27257835 10.3171/2016.3.JNS152836

[70] Julie DA , Lazow SP, Vanderbilt DB, et al. A matched-pair analysis of clinical outcomes after intracavitary cesium-131 brachytherapy versus stereotactic radiosurgery for resected brain metastases. J Neurosurg. 2020;134(5):1447–1454. 32413856 10.3171/2020.3.JNS193419

[71] Pham A , Yondorf MZ, Parashar B, et al. Neurocognitive function and quality of life in patients with newly diagnosed brain metastasis after treatment with intra-operative cesium-131 brachytherapy: a prospective trial. J Neurooncol. 2016;127(1):63–71. 26650067 10.1007/s11060-015-2009-5

[72] Chang EL , Wefel JS, Hess KR, et al. Neurocognition in patients with brain metastases treated with radiosurgery or radiosurgery plus whole-brain irradiation: a randomised controlled trial. Lancet Oncol. 2009;10(11):1037–1044. 19801201 10.1016/S1470-2045(09)70263-3

[73] Murray KJ , Scott C, Zachariah B, et al. Importance of the mini-mental status examination in the treatment of patients with brain metastases: a report from the Radiation Therapy Oncology Group protocol 91-04. Int J Radiat Oncol Biol Phys. 2000;48(1):59–64. 10924972 10.1016/s0360-3016(00)00600-3

[74] Wernicke AG , Lazow SP, Taube S, et al. Surgical technique and clinically relevant resection cavity dynamics following implantation of cesium-131 (Cs-131) brachytherapy in patients with brain metastases. Oper Neurosurg (Hagerstown). 2016;12(1):49–60. 27774500 10.1227/NEU.0000000000000986 PMC5068574

[75] Roberts DW , Strohbehn JW, Hatch JF, Murray W, Kettenberger H. A frameless stereotaxic integration of computerized tomographic imaging and the operating microscope. J Neurosurg. 1986;65(4):545–549. 3531430 10.3171/jns.1986.65.4.0545

[76] Gerard IJ , Kersten-Oertel M, Petrecca K, Sirhan D, Hall JA, Collins DL. Brain shift in neuronavigation of brain tumors: a review. Med Image Anal. 2017;35:403–420. 27585837 10.1016/j.media.2016.08.007

[77] Cao A , Thompson RC, Dumpuri P, et al. Laser range scanning for image-guided neurosurgery: Investigation of image-to-physical space registrations: laser range scanning: image-to-physical space registrations. Med Phys. 2008;35(4):1593–1605. 18491553 10.1118/1.2870216 PMC2811558

[78] Golfinos JG , Fitzpatrick BC, Smith LR, Spetzler RF. Clinical use of a frameless stereotactic arm: results of 325 cases. J Neurosurg. 1995;83(2):197–205. 7616261 10.3171/jns.1995.83.2.0197

[79] Garber ST , Jensen RL. Image guidance for brain metastases resection. Surg Neurol Int. 2012;3(Suppl 2):S111–S117. 22826814 10.4103/2152-7806.95422 PMC3400496

[80] Schackert G , Steinmetz A, Meier U, Sobottka SB. Surgical management of single and multiple brain metastases: results of a retrospective study. Onkologie. 2001;24(3):246–255. 11455217 10.1159/000055087

[81] Tan TC , Black PM. Image-guided craniotomy for cerebral metastases: techniques and outcomes. Neurosurgery. 2007;61(1 Suppl):349–56; discussion 356. 18813155 10.1227/01.neu.0000279228.50826.88

[82] Benveniste RJ , Germano IM. Correlation of factors predicting intraoperative brain shift with successful resection of malignant brain tumors using image-guided techniques. Surg Neurol. 2005;63(6):542–8; discussion 548. 15936381 10.1016/j.surneu.2004.11.025

[83] Chandler WF , Knake JE, McGillicuddy JE, Lillehei KO, Silver TM. Intraoperative use of real-time ultrasonography in neurosurgery. J Neurosurg. 1982;57(2):157–163. 7086507 10.3171/jns.1982.57.2.0157

[84] Sastry R , Bi WL, Pieper S, et al. Applications of ultrasound in the resection of brain tumors. J Neuroimaging. 2017;27(1):5–15. 27541694 10.1111/jon.12382 PMC5226862

[85] Di Lorenzo N , Esposito V, Lunardi P, Delfini R, Fortuna A, Cantore G. A comparison of computerized tomography-guided stereotactic and ultrasound-guided techniques for brain biopsy. J Neurosurg. 1991;75(5):763–765. 1919700 10.3171/jns.1991.75.5.0763

[86] Hammoud MA , Ligon BL, elSouki R, Shi WM, Schomer DF, Sawaya R. Use of intraoperative ultrasound for localizing tumors and determining the extent of resection: a comparative study with magnetic resonance imaging. J Neurosurg. 1996;84(5):737–741. 8622145 10.3171/jns.1996.84.5.0737

[87] LeRoux PD , Winter TC, Berger MS, Mack LA, Wang K, Elliott JP. A comparison between preoperative magnetic resonance and intraoperative ultrasound tumor volumes and margins. J Clin Ultrasound. 1994;22(1):29–36. 8294574 10.1002/jcu.1870220107

[88] Serra C , Stauffer A, Actor B, et al. Intraoperative high frequency ultrasound in intracerebral high-grade tumors. Ultraschall Med. 2012;33(7):E306–E312. 23129521 10.1055/s-0032-1325369

[89] de Lima Oliveira M , Picarelli H, Menezes MR, Amorim RL, Teixeira MJ, Bor-Seng-Shu E. Ultrasonography during surgery to approach cerebral metastases: effect on Karnofsky Index scores and tumor volume. World Neurosurg. 2017;103:557–565. 28359927 10.1016/j.wneu.2017.03.087

[90] Kanno H , Ozawa Y, Sakata K, et al. Intraoperative power Doppler ultrasonography with a contrast-enhancing agent for intracranial tumors. J Neurosurg. 2005;102(2):295–301. 15739558 10.3171/jns.2005.102.2.0295

[91] Engelhardt M , Hansen C, Eyding J, et al. Feasibility of contrast-enhanced sonography during resection of cerebral tumours: initial results of a prospective study. Ultrasound Med Biol. 2007;33(4):571–575. 17337111 10.1016/j.ultrasmedbio.2006.10.007

[92] Prada F , Perin A, Martegani A, et al. Intraoperative contrast-enhanced ultrasound for brain tumor surgery. Neurosurgery. 2014;74(5):542–52; discussion 552. 24598809 10.1227/NEU.0000000000000301

[93] Scholz M , Noack V, Pechlivanis I, et al. Vibrography during tumor neurosurgery. J Ultrasound Med. 2005;24(7):985–992. 15972713 10.7863/jum.2005.24.7.985

[94] Selbekk T , Bang J, Unsgaard G. Strain processing of intraoperative ultrasound images of brain tumours: initial results. Ultrasound Med Biol. 2005;31(1):45–51. 15653230 10.1016/j.ultrasmedbio.2004.09.011

[95] Chauvet D , Imbault M, Capelle L, et al. In vivo measurement of brain tumor elasticity using intraoperative shear wave elastography. Ultraschall Med. 2016;37(6):584–590. 25876221 10.1055/s-0034-1399152

[96] Prada F , Del Bene M, Rampini A, et al. Intraoperative strain elastosonography in brain tumor surgery. Oper Neurosurg (Hagerstown). 2019;17(2):227–236. 30496587 10.1093/ons/opy323

[97] Riccabona M , Nelson TR, Weitzer C, Resch B, Pretorius DP. Potential of three-dimensional ultrasound in neonatal and paediatric neurosonography. Eur Radiol. 2003;13(9):2082–2093. 12928958 10.1007/s00330-003-1845-4

[98] Unsgaard G , Ommedal S, Muller T, Gronningsaeter A, Nagelhus Hernes TA. Neuronavigation by intraoperative three-dimensional ultrasound: initial experience during brain tumor resection. Neurosurgery. 2002;50(4):804–12; discussion 812. 11904032 10.1097/00006123-200204000-00022

[99] Unsgaard G , Selbekk T, Brostrup Müller T, et al. Ability of navigated 3D ultrasound to delineate gliomas and metastases–comparison of image interpretations with histopathology. Acta Neurochir (Wien). 2005;147(12):1259–69; discussion 1269. 16172831 10.1007/s00701-005-0624-1

[100] Rygh OM , Nagelhus Hernes TA, Lindseth F, Selbekk T, Brostrup Müller T, Unsgaard G. Intraoperative navigated 3-dimensional ultrasound angiography in tumor surgery. Surg Neurol. 2006;66(6):581–92; discussion 592. 17145316 10.1016/j.surneu.2006.05.060

[101] Tronnier VM , Bonsanto MM, Staubert A, Knauth M, Kunze S, Wirtz CR. Comparison of intraoperative MR imaging and 3D-navigated ultrasonography in the detection and resection control of lesions. Neurosurg Focus. 2001;10(2):E3. 10.3171/foc.2001.10.2.4 16749750

[102] Prada F , Del Bene M, Mattei L, et al. Preoperative magnetic resonance and intraoperative ultrasound fusion imaging for real-time neuronavigation in brain tumor surgery. Ultraschall Med. 2015;36(2):174–186. 25429625 10.1055/s-0034-1385347

[103] Xiao Y , Eikenes L, Reinertsen I, Rivaz H. Nonlinear deformation of tractography in ultrasound-guided low-grade gliomas resection. Int J Comput Assist Radiol Surg. 2018;13(3):457–467. 29299739 10.1007/s11548-017-1699-x

[104] Gerard IJ , Kersten-Oertel M, Drouin S, et al. Combining intraoperative ultrasound brain shift correction and augmented reality visualizations: a pilot study of eight cases. J Med Imaging (Bellingham). 2018;5(2):021210. 29392162 10.1117/1.JMI.5.2.021210 PMC5786643

[105] Gerard IJ , Kersten-Oertel M, Hall JA, Sirhan D, Collins DL. Brain shift in neuronavigation of brain tumors: an updated review of intra-operative ultrasound applications. Front Oncol. 2020;10:618837. 33628733 10.3389/fonc.2020.618837 PMC7897668

[106] Tharin S , Golby A. Functional brain mapping and its applications to neurosurgery. Neurosurgery. 2007;60(4 Suppl 2):185–201; discussion 201. 17415154 10.1227/01.NEU.0000255386.95464.52

[107] Kombos T , Suess O, Kern BC, et al. Comparison between monopolar and bipolar electrical stimulation of the motor cortex. Acta Neurochir (Wien). 1999;141(12):1295–1301. 10672300 10.1007/s007010050433

[108] Wood CC , Spencer DD, Allison T, McCarthy G, Williamson PD, Goff WR. Localization of human sensorimotor cortex during surgery by cortical surface recording of somatosensory evoked potentials. J Neurosurg. 1988;68(1):99–111. 3275756 10.3171/jns.1988.68.1.0099

[109] Skirboll SS , Ojemann GA, Berger MS, Lettich E, Winn HR. Functional cortex and subcortical white matter located within gliomas. Neurosurgery. 1996;38(4):678–84; discussion 684. 8692384

[110] Gogos AJ , Young JS, Morshed RA, Hervey-Jumper SL, Berger MS. Awake glioma surgery: technical evolution and nuances. J Neurooncol. 2020;147(3):515–524. 32270374 10.1007/s11060-020-03482-z

[111] De Witt Hamer PC , Robles SG, Zwinderman AH, Duffau H, Berger MS. Impact of intraoperative stimulation brain mapping on glioma surgery outcome: a meta-analysis. J Clin Oncol. 2012;30(20):2559–2565. 22529254 10.1200/JCO.2011.38.4818

[112] Suarez-Meade P , Marenco-Hillembrand L, Prevatt C, et al. Awake vs. asleep motor mapping for glioma resection: a systematic review and meta-analysis. Acta Neurochir (Wien). 2020;162(7):1709–1720. 32388682 10.1007/s00701-020-04357-y

[113] Gerritsen JKW , Arends L, Klimek M, Dirven CMF, Vincent AJE. Impact of intraoperative stimulation mapping on high-grade glioma surgery outcome: a meta-analysis. Acta Neurochir (Wien). 2019;161(1):99–107. 30465276 10.1007/s00701-018-3732-4 PMC6331492

[114] Krieg SM , Schäffner M, Shiban E, et al. Reliability of intraoperative neurophysiological monitoring using motor evoked potentials during resection of metastases in motor-eloquent brain regions: clinical article. J Neurosurg. 2013;118(6):1269–1278. 23521547 10.3171/2013.2.JNS121752

[115] Krieg SM , Shiban E, Droese D, et al. Predictive value and safety of intraoperative neurophysiological monitoring with motor evoked potentials in glioma surgery. Neurosurgery. 2012;70(5):1060–70; discussion 1070. 22067415 10.1227/NEU.0b013e31823f5ade

[116] Obermueller T , Schaeffner M, Shiban E, et al. Intraoperative neuromonitoring for function-guided resection differs for supratentorial motor eloquent gliomas and metastases. BMC Neurol. 2015;15:211. 26487091 10.1186/s12883-015-0476-0 PMC4618356

[117] Chua TH , See AAQ, Ang BT, King NKK. Awake craniotomy for resection of brain metastases: a systematic review. World Neurosurg. 2018;120:e1128–e1135. 30205215 10.1016/j.wneu.2018.08.243

[118] Krieg SM , Picht T, Sollmann N, et al. Resection of motor eloquent metastases aided by preoperative nTMS-based motor maps-comparison of two observational cohorts. Front Oncol. 2016;6:261. 28066717 10.3389/fonc.2016.00261 PMC5174728

[119] Nossek E , Korn A, Shahar T, et al. Intraoperative mapping and monitoring of the corticospinal tracts with neurophysiological assessment and 3-dimensional ultrasonography-based navigation. Clinical article. J Neurosurg. 2011;114(3):738–746. 20799862 10.3171/2010.8.JNS10639

[120] Policicchio D , Ticca S, Dipellegrini G, Doda A, Muggianu G, Boccaletti R. Multimodal surgical management of cerebral lesions in motor-eloquent areas combining Intraoperative 3D Ultrasound with Neurophysiological mapping. J Neurol Surg Part Cent Eur Neurosurg. 2021;82(4):344–356. 10.1055/s-0040-1717111 33352612

[121] Cappabianca P , Cinalli G, Gangemi M, et al Application of neuroendoscopy to intraventricular lesions. Neurosurgery. 2008;62(Suppl 2):575–597; discussion 597-598. 18596446 10.1227/01.neu.0000316262.74843.dd

[122] Plaha P , Livermore LJ, Voets N, Pereira E, Cudlip S. Minimally invasive endoscopic resection of intraparenchymal brain tumors. World Neurosurg. 2014;82(6):1198–1208. 25084167 10.1016/j.wneu.2014.07.034

[123] Ma R , Coulter CA, Livermore LJ, Voets NL, Al Awar O, Plaha P. Endoscopy in temporal lobe Glioma and metastasis resection: is there a role? World Neurosurg. 2018;117:e238–e251. 29902607 10.1016/j.wneu.2018.06.006

[124] Barkhoudarian G , Farahmand D, Louis RG, et al. Microsurgical endoscope-assisted gravity-aided transfalcine approach for contralateral metastatic deep medial cortical tumors. Oper Neurosurg (Hagerstown). 2017;13(6):724–731. 29186601 10.1093/ons/opx067

[125] Zacharia BE , Romero FR, Rapoport SK, Raza SM, Anand VK, Schwartz TH. Endoscopic endonasal management of metastatic lesions of the anterior skull base: case series and literature review. World Neurosurg. 2015;84(5):1267–1277. 26079759 10.1016/j.wneu.2015.05.061

[126] Wilson DA , Duong H, Teo C, Kelly DF. The supraorbital endoscopic approach for tumors. World Neurosurg. 2014;82(6 Suppl):S72–S80. 25496639 10.1016/j.wneu.2014.07.029

[127] McLaughlin N , Prevedello DM, Engh J, Kelly DF, Kassam AB. Endoneurosurgical resection of intraventricular and intraparenchymal lesions using the port technique. World Neurosurg. 2013;79(2 Suppl):S18.e1–S18.e8. 10.1016/j.wneu.2012.02.022 22381841

[128] McLaughlin N , Filho L, Prevedello D, Kelly D, Carrau R, Kassam A. Side-cutting aspiration device for endoscopic and microscopic tumor removal. Skull Base. 2011;21(S 02):e2–e2. 10.1055/s-0032-1304834 PMC3424025 23372990

[129] Ricciardi L , Chaichana KL, Cardia A, et al The exoscope in neurosurgery: an innovative “point of view”. A systematic review of the technical, surgical, and educational aspects. World Neurosurg. 2019;124:136–144. 10.1016/j.wneu.2018.12.202 30660891

[130] Mamelak AN , Nobuto T, Berci G. Initial clinical experience with a high-definition exoscope system for microneurosurgery. Neurosurgery. 2010;67(2):476–483. 20644436 10.1227/01.NEU.0000372204.85227.BF

[131] Roethe AL , Landgraf P, Schröder T, Misch M, Vajkoczy P, Picht T. Monitor-based exoscopic 3D4k neurosurgical interventions: a two-phase prospective-randomized clinical evaluation of a novel hybrid device. Acta Neurochir (Wien). 2020;162(12):2949–2961. 32424568 10.1007/s00701-020-04361-2 PMC7593287

[132] Oertel JM , Burkhardt BW. Vitom-3D for exoscopic neurosurgery: initial experience in cranial and spinal procedures. World Neurosurg. 2017;105:153–162. 28559068 10.1016/j.wneu.2017.05.109

[133] Marenco-Hillembrand L , Suarez-Meade P, Chaichana KL. Bur hole–based resections of intrinsic brain tumors with exoscopic visualization. J Neurol Surg Part Cent Eur Neurosurg. 2021;82(02):105–111. 10.1055/s-0040-1719108 33352611

[134] Muto J , Mine Y, Nakagawa Y, et al. Intraoperative real-time near-infrared optical imaging for the identification of metastatic brain tumors via microscope and exoscope. Neurosurg Focus. 2021;50(1):E11. 10.3171/2020.10.FOCUS20767 33386024

[135] Teng CW , Cho SS, Singh Y, et al Second window ICG predicts gross-total resection and progression-free survival during brain metastasis surgery [Published online ahead of print February 2021]. J Neurosurg. 2021;135:1026–1035. doi: 10.3171/2020.8.JNS201810. 10.3171/2020.8.JNS201810 PMC10998541 33652417

[136] Moore GE , Peyton WT. The clinical use of fluorescein in neurosurgery; the localization of brain tumors. J Neurosurg. 1948;5(4):392–398. 18872412 10.3171/jns.1948.5.4.0392

[137] Stummer W , Pichlmeier U, Meinel T, Wiestler OD, Zanella F, Reulen HJ; ALA-Glioma Study Group. Fluorescence-guided surgery with 5-aminolevulinic acid for resection of malignant glioma: a randomised controlled multicentre phase III trial. Lancet Oncol. 2006;7(5):392–401. 16648043 10.1016/S1470-2045(06)70665-9

[138] Coburger J , Engelke J, Scheuerle A, et al. Tumor detection with 5-aminolevulinic acid fluorescence and Gd-DTPA-enhanced intraoperative MRI at the border of contrast-enhancing lesions: a prospective study based on histopathological assessment. Neurosurg Focus. 2014;36(2):E3. 10.3171/2013.11.FOCUS13463 24484256

[139] Kamp MA , Munoz-Bendix C, Mijderwijk HJ, et al. Is 5-ALA fluorescence of cerebral metastases a prognostic factor for local recurrence and overall survival? J Neurooncol. 2019;141(3):547–553. 30535595 10.1007/s11060-018-03066-y

[140] Hussein A , Rohde V, Wolfert C, et al. Survival after resection of brain metastases with white light microscopy versus fluorescence-guidance: A matched cohort analysis of the Metastasys study data. Oncotarget. 2020;11(32):3026–3034. 32850007 10.18632/oncotarget.27688 PMC7429181

[141] Schebesch KM , Proescholdt M, Höhne J, et al. Sodium fluorescein-guided resection under the YELLOW 560 nm surgical microscope filter in malignant brain tumor surgery–a feasibility study. Acta Neurochir (Wien). 2013;155(4):693–699. 23430234 10.1007/s00701-013-1643-y

[142] Schebesch KM , Hoehne J, Hohenberger C, et al. Fluorescein sodium-guided resection of cerebral metastases—experience with the first 30 patients. Acta Neurochir (Wien). 2015;157(6):899–904. 25824557 10.1007/s00701-015-2395-7

[143] Höhne J , Hohenberger C, Proescholdt M, et al. Fluorescein sodium-guided resection of cerebral metastases-an update. Acta Neurochir (Wien). 2017;159(2):363–367. 28012127 10.1007/s00701-016-3054-3

[144] Xiao SY , Zhang J, Zhu ZQ, et al. Application of fluorescein sodium in breast cancer brain-metastasis surgery. Cancer Manag Res. 2018;10:4325–4331. 30349366 10.2147/CMAR.S176504 PMC6190807

[145] Falco J , Cavallo C, Vetrano IG, et al. Fluorescein application in cranial and spinal tumors enhancing at preoperative MRI and operated with a dedicated filter on the surgical microscope: preliminary results in 279 patients enrolled in the FLUOCERTUM prospective study. Front Surg. 2019;6:49. 31475153 10.3389/fsurg.2019.00049 PMC6705221

[146] Hamamcıoğlu MK , Akçakaya MO, Göker B, Kasımcan MÖ, Kırış T. The use of the YELLOW 560 nm surgical microscope filter for sodium fluorescein-guided resection of brain tumors: Our preliminary results in a series of 28 patients. Clin Neurol Neurosurg. 2016;143:39–45. 26895208 10.1016/j.clineuro.2016.02.006

[147] Kofoed MS , Pedersen CB, Schulz MK, et al Fluorescein-guided resection of cerebral metastases is associated with greater tumor resection [Published online March 15 2021]. Acta Neurochir (Wien). 2021. doi:10.1007/s00701-021-04796. 10.1007/s00701-021-04796-1 33721109

[148] Zhang DY , Singhal S, Lee JYK. Optical principles of fluorescence-guided brain tumor surgery: a practical primer for the neurosurgeon. Neurosurgery. 2019;85(3):312–324. 30085129 10.1093/neuros/nyy315 PMC12311972

[149] Lee JY , Thawani JP, Pierce J, et al. Intraoperative near-infrared optical imaging can localize gadolinium-enhancing Gliomas during surgery. Neurosurgery. 2016;79(6):856–871. 27741220 10.1227/NEU.0000000000001450 PMC5123788

[150] Lee JYK , Pierce JT, Zeh R, et al. Intraoperative near-infrared optical contrast can localize brain metastases. World Neurosurg. 2017;106:120–130. 28669877 10.1016/j.wneu.2017.06.128 PMC11073792

